# Supplementary material for: Tryptophan Metabolism Is Associated with BMI and Adipose Tissue Mass and Linked to Metabolic Disease in Pediatric Obesity
Source: Nutrients. 2022 Jan 11;14(2):286. doi: 10.3390/nu14020286 (PMC8781866; doi:10.3390/nu14020286)
Supplement: Supplementary file 1 [file nutrients-14-00286-s001.zip › nutrients-1484064-supplementary.pdf]

Supplementary Table S1. Distribution of the study cohort among MHO criteria.

MHO Criteria	Count	Percent
HDL-C > 40 mg/dl	73	58.90%
Triglycerides ≤ 150 mg/dl	95	76.60%
Fasting glucose ≤ 100 mg/dl	116	93.50%
Systolic and diastolic blood pressure ≤ 90th percentile	76	59.80%
